# The *in vitro* influences of epidermal growth factor and heregulin-β1 on the efficacy of trastuzumab used in Her-2 positive breast adenocarcinoma

**DOI:** 10.1186/1475-2867-13-97

**Published:** 2013-10-11

**Authors:** Tracey Hurrell, Kim Outhoff

**Affiliations:** 1 Department of Pharmacology, Faculty of Health Sciences, School of Medicine, University of Pretoria, Private Bag X323, Pretoria 0007, South Africa

**Keywords:** Her-2 receptors, Trastuzumab, EGF, Heregulin-β1, SK-Br-3 cells

## Abstract

**Background:**

Human epidermal growth factor receptor-2 (Her-2) is over expressed in approximately 25-30% of all primary breast tumors resulting in a distinctive breast cancer subtype associated with a poor prognosis and a decrease in overall survival. Trastuzumab (Herceptin®), an anti-Her-2 monoclonal antibody, has dramatically altered the prognosis of Her-2 positive breast cancer. Trastuzumab is, however, associated with primary and acquired resistance.

**Aim and methods:**

To investigate the *in-vitro* effects of trastuzumab on cell viability (tetrazolium conversion assay), cell cycling (propidium iodide staining), apoptosis (executioner caspases and annexin-V) and relative surface Her-2 receptor expression (anti-Her-2 affibody molecule) in Her-2-positive (SK-Br-3) and oestrogen receptor positive (MCF-7) breast adenocarcinoma cells and to determine potential augmentation of these effects by two endogenous ligands, epidermal growth factor (EGF) and heregulin-β1 (HRG- β1).

**Results:**

Cell viability was decreased in SK-Br-3 cells by exposure to trastuzumab. This was associated with G1 accumulation and decreased relative surface Her-2 receptor density, supporting the cytostatic nature of trastuzumab *in vitro*. SK-Br-3 cells exposed to EGF and heregulin-β1 produced differential cell responses alone and in combination with trastuzumab, in some instances augmenting cell viability and cell cycling. Relative surface Her-2 receptor density was reduced substantially by trastuzumab, EGF and heregulin-β1. These reductions were amplified when ligands were used in combination with trastuzumab.

**Conclusion:**

Cell type specific interactions of endogenous ligands appear to be dependent on absolute Her-receptor expression and cross activation of signaling pathways. This supports the notion that receptor density of Her-family members and multiplicity of growth ligands are of mutual importance in breast cancer cell proliferation and therefore also in resistance associated with trastuzumab.

## Background

The evolutionary ancient human epidermal growth factor receptor (Her) family is composed of the structurally related tyrosine kinases Her-1 (EGFR), Her-2, Her-3 and Her-4, which play a fundamental role in regulating cell functions including proliferation, adhesion, motility and survival [1-3]. Her-family receptors govern different functions and have different properties. Furthermore, Her-1, Her-3 and Her-4 possess the affinity for binding multiple ligands [4,5]. Ligands for Her-1 include EGF, transforming growth factor-α, amphiregulin, betacellulin, epigen, epiregulin and heparin binding EGF-like growth factor while ligands for Her-3 and Her-4 include the isoforms of four structurally related heregulins [5].

In contrast with the others, Her-2 receptors have no known ligands and are thus designated as orphan receptors [6-8]. Rather, activation of these receptors is achieved by the formation of interactive dimers, either spontaneously with Her-2 receptors or with other ligand-activated family members. Her-3 receptors are incapable of intrinsic kinase activity [9]; thus ordinarily, neither Her-2 nor Her-3 are capable of linear signaling in isolation, which implies that they have strongly interdependent signaling characteristics [5].

Modularity, redundancy and the capacity for combination interactions are important in signal diversification of the Her-signaling network [6]. These horizontal networks can be detrimental when over expressed receptors spontaneously homo-dimerize or bias the dimer types formed [10]. Equally, over-production of endogenous ligands such as EGF and heregulin-β1 may over-activate these networks as part of the carcinogenesis process [11,12]. Approximately 25-30% of all primary breast tumors over express Her-2 receptors, [11] which makes the Her-2 receptor a clinically relevant molecular constituent of breast cancer associated with a poor prognosis and a decrease in overall survival [13].

Trastuzumab (Herceptin®; Genentech Inc, South San Francisco, CA), is a recombinant, DNA derived, humanized, anti-Her-2 monoclonal antibody which selectively targets subdomain IV of the extracellular domain over expressed Her-2 receptors and is licensed as a therapy for Her-2 positive breast cancer.

Trastuzumab, while governed by strict eligibility criteria, has become an integral component of treatment regimens and has dramatically altered the natural progression of this breast cancer subtype [14,15]. Initially, studies reported that in trastuzumab-treated patients, Her-2 status remained stable over time. However, discordances between primary and metastatic sites are now reported to reach up to 30%. This dynamic receptor expression confounds stratification of patients into appropriate therapeutic categories [16].

Furthermore, despite having enhanced selectivity for Her-2 over expressing cancerous cells, [17] trastuzumab efficacy is hampered by significant variations in response and the development of resistance [18,19]. Retrospective analyses with the paradigm of targeted therapies in other cancer subtypes (such as non-small-cell lung cancer) suggest that only 30-40% of patients derive substantial clinical benefit from molecular targeted therapies and that long term disease remission is not achieved in up to 80% of cancer patients [20,21].

Recent data from the Hermine observational study, designed to evaluate outcomes in patients with metastatic breast cancer receiving trastuzumab in routine clinical practice, illustrated that while survival benefit was evident for patients on first line trastuzumab treatment, 177 (80.09%) patients from the cohort experienced disease progression within the follow up period [22].

The aim of this study, therefore, was to investigate the ability of the endogenous Her-receptor activating ligands EGF (Her-1 ligand) [5] and heregulin- β1 (Her-3 and Her-4 ligand) [23,24] to influence the efficacy of trastuzumab in Her-2 positive breast adenocarcinoma in order to gain a clearer understanding of what may occur when the biological context is altered.

## Results and discussion

Tight integration and redundancy of the Her-receptor system creates contemporary challenges for targeted therapies such as trastuzumab, including acquired and *de novo* resistance [25] associated with, among others, truncated Her-2 (p95-Her-2) forms, repackaging of integral cell survival proteins and up regulation of alternative signalling pathways [19,26]. Here we discuss the ability of two endogenous molecules to alter *in vitro* characteristics of trastuzumab and assess the implications for targeted therapy.

**Figure 1:**
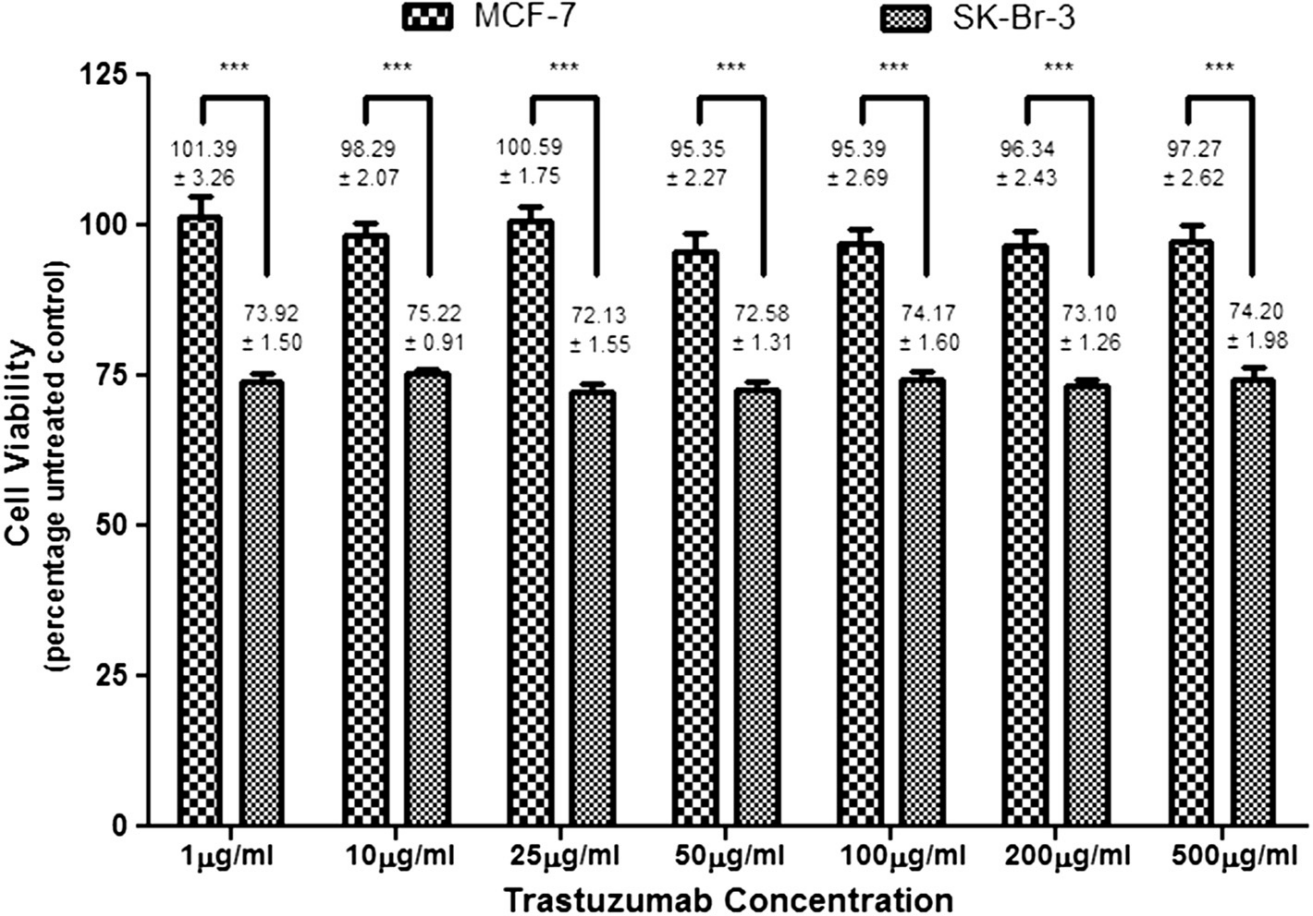
**Cell viability in MCF-7 and SK-Br-3 cells.** Mean (± SEM) cell viability determined using a tetrazolium conversion assay. Cells were first exposed to trastuzumab ranging from 1 μg/ml to 500 μg/ml. The result was a reduction in cell viability in SK-Br-3 cells which was not seen in MCF-7 cells. Cells were then exposed to a saturating concentration of trastuzumab combined with either EGF (400 ηg/ml) or HRG-β1 (200 ηg/ml). These endogenous biological molecules significantly abrogated cell viability observed for trastuzumab (Table 1).

The greater the dependence of cells on Her-2 receptor mediated growth and transformation, the greater the anti-proliferative effect of trastuzumab [27]. Not surprisingly, trastuzumab was unable to affect cell viability in MCF-7 cells. Trastuzumab (1–500 μg/ml) induced substantial anti-proliferative effects in SK-Br-3 cells however (Figure 1), which suggests an extensive reliance of these cells on Her-2 receptors for propagation of cell growth [28]. However, there was no difference in cell viability at different concentrations of trastuzumab. It was speculated that at higher concentrations, the binding domains were saturated or that Her-2 receptors were internalised and degraded or unable to recycle back to the surface for further trastuzumab binding. Saturating concentrations of trastuzumab (50 – 100 μg/ml) were used for further studies to ensure continuous effects on the seeded cell population as well as on cell progeny.

**Figure 2:**
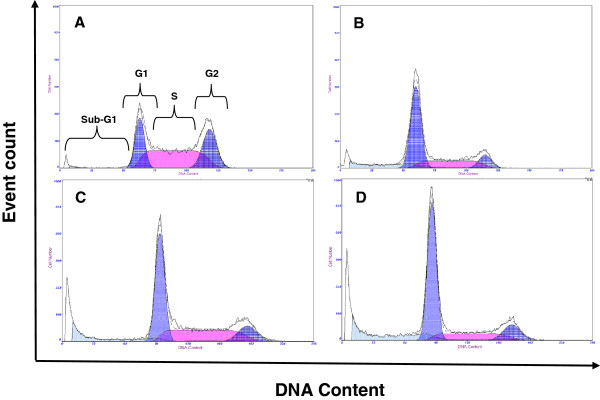
**Flow cytometry histograms analysed with deconvolution software.** Gaussian curves were utilised to divide histograms into G1 phase (left), S phase (centre) and G2 phase (right) **A)** Untreated MCF-7 cells in DMEM with 10% FCS; **B)** Trastuzumab treated MCF-7 cells after 72 hours illustrating G1 accumulation; **C)** Untreated SK-Br-3 cells in RPMI with 10% FCS; **D)** Trastuzumab treated SK-Br-3 cells after 24 hours illustrating G1 accumulation.

**Figure 3:**
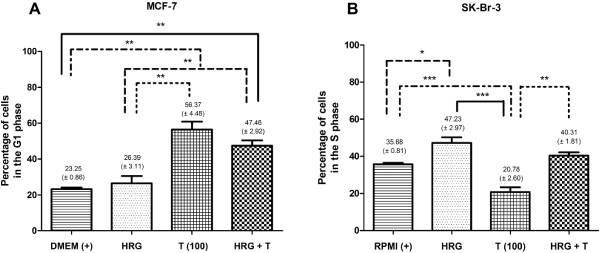
**Column graphs of cell cycle analysis.** Following exposure for 72 hours to trastuzumab, heregulin-β1 or the heregulin-β1-trastuzumab combination histograms were analyzed with deconvolution software and expressed as column graphs. **A)** Significant alterations within the G1 phase were observed in MCF-7 cells exposed to trastuzumab and the heregulin-β1-trastuzumab combination; **B)** Significant alterations in the S-phase were observed in SK-Br-3 cells exposed to heregulin-β1 compared to the untreated control. [EGF did not perpetuate noteworthy accumulation or alterations in cell cycle kinetics].

Trastuzumab influenced cell cycle kinetics by inducing significant G1 accumulation in MCF-7 at 72 hours only (Figures 2 and 3) and in SK-Br-3 cells at 24, 48 (data not shown) and 72 hours (Figure 2). This is consistent with other data where trastuzumab has been found to interfere with Her-2 receptor signaling and consequently inhibit G1-S phase transition [29,30].

**Figure 4:**
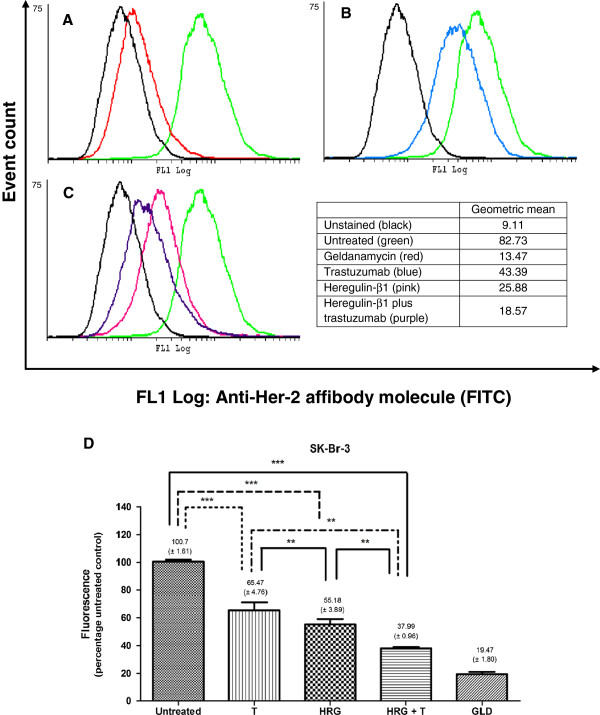
**Overlay plots and column graphs comparing the relative Her-2 receptor density in SK-Br-3 cells. A)** Overlay plots of the unstained (black), stained (green) and the positive control, geldanamycin (red) with the associated geometric mean; **B)** Overlay plots of the unstained (black), stained (green) and cells exposed to trastuzumab (blue) with the associated geometric mean; **C)** Overlay plots of the unstained (black), stained (green) and cells exposed to heregulin-β1 (pink) or the heregulin-β1-trastuzumab combination (purple) with the associated geometric mean; **D)** Fluorescence (x-mean) intensity of triplicate runs, expressed as a percentage of untreated control (standardised to 100%). Concurrent heregulin-β1 abrogated relative Her-2 receptor density observed for trastuzumab. Trastuzumab, heregulin-β1 and the heregulin-β1-trastuzumab combination illustrated a progressive decrease in Her-2 receptor density at 24 hours. Similar significance trends were observed in SK-Br-3 cells exposed to EGF [56.82% (± 2.82)] and the EGF-trastuzumab combination [45.01% (± 1.06)].

While targeted therapies have achieved great commendation for their selectivity and specificity, efficacy remains dependent on the continual and persistent presence of targets. Unlike MCF-7 cells, which were impervious to the effects of trastuzumab, SK-Br-3 cells demonstrated a significant decrease in Her-2 receptors when exposed to trastuzumab from as early as 6 hours [68.69% ± (1.14)]. Near identical decreases were obtained at 12, 24 (Figure 4) and 48 hours suggesting that cells were not re-accumulating on the surface within the period of time assessed. Taken together, the dose-independent decrease in cell viability and consistent G1 accumulation in SK-Br-3 cells may have been due to the reduction in Her-2 receptors, a threshold above which is required for further trastuzumab efficacy.

Binding of Her-receptor ligands results in the formation of dimer complexes. Subsequent auto-phosphorylation results in intracellular signal propagation promoting cell proliferation [31]. The pleiotropic biological activity of Her-ligands creates doubt as to the ability of targeted therapies to elicit clinically valuable responses in the presence of these ligands. Exposing MCF-7 cells to EGF (400 ηg/ml) or heregulin-β1 (200 ηg/ml) resulted in inconsequential responses. However, both ligands appeared to sensitize MCF-7 cells to the anti-proliferative effects of trastuzumab: when cells were exposed to the combination of growth factors and trastuzumab, a significant decrease in viability was noted, compared to each agent used alone. (Table 1). These reductions, when viewed in isolation may suggest that trastuzumab may influence Her-2 negative, estrogen receptor positive breast cancer, particularly in the presence of overexpressed Her-family ligands.

**Table 1:** Cell viability in MCF-7 and SK-Br-3 cells

	**MCF-7**	**SK-Br-3**
Trastuzumab *(50 μg/ml)*	95.35% (± 2.27)	72.58% (± 1.31)
	**(EGF + T**_**50**_**: P < 0.001)**	
EGF *(400 ηg/ml)*	101.70% (± 1.84)	81.43% (± 2.46)
	**(EGF + T**_**50**_**: P < 0.001)**	
EGF *(400 ηg/ml)* + Trastuzumab *(50 μg/ml)*	89.52% (± 1.95)	89.40% (± 2.20)
		**(Trastuzumab**_**50**_**: P < 0.05)**
HRG *(200 ηg/ml)*	107.01% (± 2.42)	115.40% (± 2.08)
	**(Trastuzumab**_**50**_**: P < 0.001)**	**(Trastuzumab**_**50**_**: P < 0.001)**
HRG *(200 ηg/ml)* + Trastuzumab *(50 μg/ml)*	93.76% (±2.52)	114.50% (± 1.98)
	**(HRG: P < 0.001)**	**(Trastuzumab**_**50**_**: P < 0.001)**

Mean (± SEM) cell viability determined using a tetrazolium conversion assay.

This correlates with others who have demonstrated trastuzumab’s ability to inhibit heregulin-induced proliferation of these cells, in spite of normal expression of Her-2 receptors [32,33]. However, in these experiments, trastuzumab merely diminished the enhanced cell viability induced by Her-family ligands, which seldom have altered expression levels in Her-2 negative breast cancer subtypes. It does however highlight the ability to manipulate signaling pathways of subtypes of breast cancer based on the presence of endogenous ligands and suggests that our scope of clinical assessments is too narrow to confer positive clinical outcomes in circumstances confounded by endogenous ligands. Further elucidation of molecular crosstalk between oestrogen and Her-family growth signalling pathways which become activated under various physiological conditions by endogenous ligands is integral in determining therapeutic strategies.

That no apoptosis (via detection of executioner caspases or annexin-V) was detected for any agent used alone or in combination with trastuzumab despite significant apoptosis being observed for the positive control (data not shown), suggested that mechanisms for altered *in vitro* cell viability were cytostatic, possibly due to alterations in cell cycle kinetics, rather than being cytotoxic. EGF, a proliferative Her-1 ligand, should theoretically accelerate cell cycle kinetics and increase the number of cells entering the G1 phase. However, in this study, EGF exposure resulted in negligible effects on the cell cycle of MCF-7 cells, which correlated with the lack of additional cell growth. Notably, G1 phase accumulation was observed in the cells exposed to the EGF-trastuzumab combination [38.54% (± 1.25)] and while increased compared to the control, G1 accumulation remained statistically significantly lower than trastuzumab.

Heregulin binding assays have quantified Her-3 and Her-4 receptors and indicate that SK-Br-3 cells express more than double the number of Her-3 and Her-4 receptors per cell than MCF-7 cells and are thus arbitrarily characterized as having intermediate and high levels of receptors respectively [34]. In MCF-7 cells, heregulin demonstrated no cumulative effects on cell cycle distribution (Figure 3). However, at 24 hours, a slight G2 phase increase was noted, suggesting accelerated cell cycle kinetics to accompany the increased cell viability.

Her-2 monoclonal antibodies are capable of inhibiting heregulin-induced activation of PI3-kinase and downstream targets (Akt) in MCF-7 cells [23]. This inhibition could be the mechanism by which trastuzumab abrogated heregulin-induced mitogenic proliferation in our cells. The accompanying G1 accumulation in cells exposed to a combination of heregulin and trastuzumab, which mimicked that of trastuzumab, may also be attributed to inhibition of this pathway. Although we were unable to observe alterations in Her-2 receptor density, perhaps due to the low constitutive Her-2 receptor number of MCF-7 cells, the presence of these endogenous ligands appeared to influence the mechanisms that we and others have assessed for trastuzumab in MCF-7 cells.

Although mRNA can be a deceptive framework for referencing ligand efficacy, quantitative analysis shows that SK-Br-3 cells express approximately ten-times more Her-1 protein than MCF-7 cells [35] which implies a greater potential for response to EGF. However, EGF resulted in a surprising decrease in cell viability of SK-Br-3 cells. This was counteracted by concurrent trastuzumab exposure in favor of increased cell viability (Table 1). Synergistic effects of over expressing receptors within cellular transformation have been suggested [24]. However, sustained activation of Her-1 and Her-2 receptors may activate pathways, paradoxically leading to cell death even in the presence of proliferative ligands [36,37].

EGF exposure resulted in a substantial decrease in Her-2 receptor density, implying that Her-2 signaling was reduced. When combined with trastuzumab, the decline in Her-2 receptor density was even greater. Tikhomirov *et al.* noted that rapid internalization of Her-1 and subsequent lysosomal degradation occurs after ligand binding [37]; Her-2 receptors may be internalized as part of this interactive dimer complex. Here the authors suggest that reduction of Her-2 receptors by trastuzumab may alter the balance of Her-1:Her-2 co-expression, and in doing so, EGF potentiates less of an anti-proliferative effect. Thus in selective circumstances, where multiple Her-receptors are over-expressed, trastuzumab may alter the co-expression ratio in favor of synergistic proliferative effects.

Heregulin-β1-induced propagation of cell growth of SK-Br-3 cells (Table 1) was consistent with the fact that these cells also express Her-3 receptors, and was accompanied by an increased number of cells in the S-phase of the cell cycle at 72 hours (Figure 3). Her-2:Her-3 heterodimers are considered the most potent signal transduction complex with the highest mitogenic potential [5,17]. Increased cell growth by heregulin-β1 could also be attributed to potentiation of already increased basal Akt activity associated with Her-2 receptor over-expression [23]. Thus, as a potent stimulator, heregulin-β1 may be capable of compensating for the inhibition ordinarily elicited by trastuzumab [38]. This was corroborated when concurrent exposure with trastuzumab did not affect the cell growth potential of heregulin-β1. Once again, the potency of the Her-2:Her-3 dimer pair could help explain why trastuzumab had a negligible effect on these cells when combined with heregulin-β1.

Although heregulin-β1 propagated cell growth, substantial reductions in Her-2 receptors were observed when SK-Br-3 cells were exposed to the ligand. An even greater reduction of Her-2 receptors was observed when heregulin-β1 was used in combination with trastuzumab (Figure 4). This could have serious implications for trastuzumab efficacy, as the target is diminished. Daly *et al*. previously also observed a decrease in Her-2 receptors in response to heregulin-β1 after 48 hours in these cells [39].

Reduction in surface Her-2 receptors by EGF and heregulin-β1 implicates both growth ligands in limiting trastuzumab efficacy and ultimately resulting in disease progression. If this remains true for additional Her-family ligands, assessing expression levels of ligands will have serious therapeutic implications in the clinical paradigm of trastuzumab eligibility.

In our experiments, Her-2 receptors were assessed using whole cells because trastuzumab efficacy is solely dependent on the accessibility of receptors on the cell surface. Receptor analysis using lysed cells potentially also detects ubiquitin-targeted receptors which are not yet degraded, or internalised receptors capable of recycling; this may skew data in favour of a higher receptor density. EGF and heregulin-β1 appeared to promote Her-2 receptor internalization in the form of heterodimers which augmented the reduction of Her-2 receptor in trastuzumab exposed cells. However, the fate of receptors upon internalization was not elucidated as it was beyond the scope of this experiment. While the overall number of receptors within a cell may not change in response to trastuzumab, the ability of receptors to be restored to the cell surface upon removal of trastuzumab or ligands remains inconclusive. Further complicating this research is the ability of factors such as fetal calf serum (FCS) and seeding density to provide differential responses in endpoint experiments dependent on the heregulin isoforms used [39]. While heregulin-β1 was used in these experiments the results are intriguing and open avenues for the study of alternative isoforms. Understanding and monitoring the molecular subtypes of cancer and divergence of signaling pathways in response to targeted therapy is now required to ensure the therapeutic benefit of trastuzumab.

While the use of cytotoxic-conjugated-antibodies is being researched extensively, antibody-dependent cell-mediated cytotoxicity (ADCC) was not assessed in this study. This is due to the fact that efficacy of trastuzumab in a clinical context is highly dependent on the maintenance of a continuous and consistent receptor threshold. If this is not done, as was seen over the time points assessed here then ADCC would be negligible due to the absence of sufficient target receptors.

## Conclusion

Cell type specific interactions of endogenous ligands appear to be dependent on the absolute Her-receptor expression and cross activation of signaling pathways. While a threshold of surface Her-2 receptors is required to ensure trastuzumab binding, efficacy may be dependent on co-receptor expression ratios, as opposed to only Her-2 receptors, as a prognostic indicator and marker for targeted therapy. In this study, Her-2 receptor status was greatly influenced by endogenous growth factors. The reduction in surface Her-2 receptors diminished the availability of targets for trastuzumab and thereby differentially altered the efficacy parameters assessed for trastuzumab. It remains unclear whether these alterations in receptor abundance are maintained for sufficient periods to be clinically relevant. However, this data supports the notion that receptor density of Her-family members and multiplicity of growth ligands are of mutual importance in proliferation. This may provide insight into the role of endogenous Her-ligands in the emergence of resistance and have far-reaching consequences for targeted therapy and long-term disease remission.

## Methods

This study was approved by the Faculty of Health Science Student Research Ethics Committee of the University of Pretoria.

### Human breast adenocarcinoma cells

Adherent breast adenocarcinoma cell lines included estrogen receptor positive MCF-7 cells (ATCC Number HTB-20^TM^) which express physiologically normal levels of Her-2 receptors, and SK-Br-3 cells (ATCC Number HTB-30^TM^) which over-express Her-2 receptors, obtained from American Type Culture Collection (ATCC; Manassas, USA). MCF-7 cells were deemed an ideal control as these cells express estrogen receptors, enabling the potential for receptor crosstalk, while both MCF-7 and SK-Br-3 cells express all members of the Her-family of receptor tyrosine kinases to varying degrees. MCF-7 and SK-Br-3 cells were maintained in DMEM and RMPI 1640 medium (Sigma-Aldrich; St Louis, USA) respectively, supplemented with 10% heat-inactivated fetal calf serum (FCS) (PAA Laboratories; Pasching, Austria) and 1% penicillin-streptomycin (BioWhittaker; Walkersville, USA). Cells were seeded at 1×10^4^ cells per well or at 1.5×10^5^ cells per 25 cm^2^ flask and maintained in a humidified atmosphere containing 5% CO_2_ at 37°C.

### Trastuzumab and growth factors

Trastuzumab (Herceptin®) was kindly donated by Roche Pharmaceuticals (Johannesburg, South Africa). Recombinant human epidermal growth factor and heregulin-β1 were obtained as lyophilized powder from Sigma-Aldrich (St Louis, USA). Growth factors were reconstituted in filter sterilized (0.22 μM) dH_2_O while trastuzumab was reconstituted in bacteriostatic water as per manufacturer’s instructions. Cells were exposed to saturating concentrations of EGF (400 ηg/ml) or heregulin-β1 (200 ηg/ml) to determine baseline effects. Additionally, cells were exposed to trastuzumab (1–500 μg/ml), to determine a dose response and a saturating concentration of trastuzumab then combined with either EGF or heregulin-β1.

### Tetrazolium conversion assay

Cell viability was determined using a quantitative-colorimetric conversion assay of 3-[4, 5-dimethylthiazol-2-yl]-2, 5-diphenyl tetrazolium bromide (MTT) after exposure for 96 hours. Cells were incubated with 20 μl MTT solution (5 mg/ml) (Sigma-Aldrich; St Louis, USA) for 3 hours, washed with phosphate buffered saline (PBS) (BD Bioscience; Sparks, USA) and the formazan product solubilized in dimethyl sulphoxide (Merck Chemicals; Darmstadt, Germany). Plates were read spectophotometrically using an ELx800uv universal microplate reader (Bio-TEK Instruments, INC) with a dual wavelength of 570 nm and 630 nm. Cell-free medium and drug controls were included to discern reactivity of MTT with extraneous variables other than cells.

### Cell cycle analysis

The cell cycle was analyzed using interchelating, fluorescent propidium iodide with flow cytometric detection at 24, 48 and 72 hours. Decanted medium and trypsinized cells were washed (1% FCS in PBS), fixed in 70% ethanol (Merck Chemicals; Darmstadt, Germany) and stored overnight at 4°C. Cell pellet was re-suspended in 1 ml of propidium iodide (40 μg/ml) (Sigma-Aldrich; St Louis, USA) staining solution containing Triton-X (Sigma-Aldrich; St Louis, USA), a non-ionic surfactant (0.1% v/v), and DNase free RNase (100 μg/ml) (Sigma-Aldrich; St Louis, USA). Samples were incubated at 37°C for 40 minutes and analyzed using a Cytomics FC500 (Beckman Coulter).

### Executioner caspase assay

Cells were exposed to trastuzumab, growth factors or the positive control [staurosporine, 10.7 μM (Sigma-Aldrich; St Louis, USA)] between 4 and 30 hours. Cell lysis buffer [10 mM 4-(2-hydroxyethyl)-1-piperazineethanesulfonic acid (HEPES), 1 mM phenylmethylsulfonyl fluoride (PMSF), 5 mM 3-[(3-cholamidopropyl)dimethylammonio]-1-propanesulfonate (CHAPS) (Merck Chemicals; Darmstadt, Germany), 2 mM ethlene-diamine-tetra-acetic acid (EDTA), and 5 mM β-mercaptoethanol (Labchem; Johannesburg, RSA)] was added and plates incubated for 40 minutes on ice followed by addition of 125 μl assay buffer [20 mM HEPES, 2 mM EDTA and 5 mM β-mercaptoethanol] containing 5 μM of Ac-DEVD-AMC substrate (Sigma-Aldrich; St Louis, USA). Plates were incubated overnight at 37°C to allow Ac-DEVD-AMC substrate hydrolysis by activated executioner caspases-3 and -7, and the released fluorescent product was read using a FLUOstar OPTIMA (BMG LABTECH) with excitation-emission wavelengths of 350 nm and 450 nm respectively.

### Apoptosis-necrosis

Later apoptotic hallmarks were assessed after exposure for 48 or 72 hours using FITC conjugated annexin-V, counterstained with propidium iodide to assess membrane integrity. Cells were washed and re-suspended in 100 μl of annexin-V binding buffer [10 mM HEPES, 150 mM NaCl, 5 mM KCL, 1.8 mM CaCl_2_ and 1 mM MgCl_2_ (Merck Chemicals; Darmstadt, Germany)] followed by staining with 2.5 μl of annexin-V-FITC (Sigma-Aldrich; St Louis, USA). Samples were incubated in the dark for 10 minutes and 3 μl of propidium iodide (3 mM) (Sigma-Aldrich; St Louis, USA) was added shortly prior to acquisition using a Cytomics FC500 (Beckman Coulter).

### Relative Her-2 receptor density

Relative Her-2 receptor density was determined after cells were exposed to trastuzumab, growth factors or the positive control [geldanamycin, 0.35 μM (Tocris Bioscience; Ellisville, USA)] for 12, 24 and 48 hours, using an anti-Her-2-FITC affibody molecule (Abcam; Cambridge, UK) which is a protein composed of a three-helix bundled at the unique C-terminal cysteine which does not compete for Her-2 binding with trastuzumab [40]. Samples were washed twice (1% FCS in PBS) and the single cell suspension labeled with the affibody molecule (final concentration: 3.7 μg/ml) and incubated on ice for 30 minutes. Cells were washed prior to acquisition using a Cytomics FC500 (Beckman Coulter). The mean-fluorescence intensity was expressed relative to the untreated control.

### Statistics

A minimum of three independent inter-day repeats were conducted with a minimum of three intra-day repeats where required. Cell cycle histograms were analysed using deconvolution software, uncovering the underlying distribution by fitting Gaussian curves using WinCycle MultiCycle V3.0 for Windows (WinCycle Software; Washington, USA). Histrograms for relative Her-2 receptor density were analysed using Cyflogic^TM^ software (CyFlo, Ltd, Finland) and the x-mean and geometric mean determined. Non-parametric, Kruskal-Wallis, test was conducted to compare the mean values obtained for trastuzumab versus EGF or heregulin-β1 alone versus the trastuzumab-growth factor combinations. Dunn’s multiple comparison test was used for post-hoc analysis with significance set at p-value < 0.05. Statistical analysis was conducted using GraphPad Prism version 5.0 for Windows (GraphPad Software; San Diego, California, USA).

## Abbreviations

ADCC: Antibody-dependent cell-mediated cytotoxicity; Akt: Protein kinase B; CHAPS: 3-[(3-cholamidopropyl)dimethylammonio]-1-propanesulfonate; EDTA: Ethlene-diamine-tetra-acetic acid; EGF: Epidermal growth factor; FCS: Fetal calf serum; HEPES: 4-(2-hydroxyethyl)-1-piperazineethanesulfonic acid; Her: Human epidermal growth factor receptor; HRG-β1: Heregulin-β1; MTT: 3-[4, 5-dimethylthiazol-2-yl]-2, 5-diphenyl tetrazolium bromide; PBS: Phosphate buffered saline; PMSF: Phenylmethylsulfonyl fluoride; TTP: Time to progression.

## Competing interests

The authors declare that they have no conflict of interest, financial or non-financial, with respect to this research.

## Authors’ contributions

KO conceived the study, participated in its design and coordination, acquired funding for the study and assisted in drafting and critically revising the manuscript. TH carried out the experimental procedures, performed the analysis and assisted in drafting the manuscript. All authors read and approved the final manuscript.
